# Toward Emotion Recognition From Physiological Signals in the Wild: Approaching the Methodological Issues in Real-Life Data Collection

**DOI:** 10.3389/fpsyg.2020.01111

**Published:** 2020-07-15

**Authors:** Fanny Larradet, Radoslaw Niewiadomski, Giacinto Barresi, Darwin G. Caldwell, Leonardo S. Mattos

**Affiliations:** ^1^Advanced Robotics, Istituto Italiano di Tecnologia, Genoa, Italy; ^2^Contact Unit, Istituto Italiano di Tecnologia, Genoa, Italy; ^3^Department of Psychology and Cognitive Science, University of Trento, Rovereto, Italy; ^4^Rehab Technologies, Istituto Italiano di Tecnologia, Genoa, Italy

**Keywords:** emotion recognition, data collection, in-the-wild, physiological signals, emotion elicitation and assessment

## Abstract

Emotion, mood, and stress recognition (EMSR) has been studied in laboratory settings for decades. In particular, physiological signals are widely used to detect and classify affective states in lab conditions. However, physiological reactions to emotional stimuli have been found to differ in laboratory and natural settings. Thanks to recent technological progress (e.g., in wearables) the creation of EMSR systems for a large number of consumers during their everyday activities is increasingly possible. Therefore, datasets created in the wild are needed to insure the validity and the exploitability of EMSR models for real-life applications. In this paper, we initially present common techniques used in laboratory settings to induce emotions for the purpose of physiological dataset creation. Next, advantages and challenges of data collection in the wild are discussed. To assess the applicability of existing datasets to real-life applications, we propose a set of categories to guide and compare at a glance different methodologies used by researchers to collect such data. For this purpose, we also introduce a visual tool called Graphical Assessment of Real-life Application-Focused Emotional Dataset (GARAFED). In the last part of the paper, we apply the proposed tool to compare existing physiological datasets for EMSR in the wild and to show possible improvements and future directions of research. We wish for this paper and GARAFED to be used as guidelines for researchers and developers who aim at collecting affect-related data for real-life EMSR-based applications.

## 1. Introduction

Emotion, mood and stress recognition (EMSR)^1^ from facial expression (Fasel and Luettin, 2003), speech (El Ayadi et al., 2011), full-body motion (Kleinsmith and Bianchi-Berthouze, 2013), text (Hirschberg and Manning, 2015), and physiological signals (Jerritta et al., 2011) has been studied intensively for at least two decades. The EMSR models can be differentiated according to the emotion theory adopted to characterize the data. While using labels such as anger, disgust, fear, joy, sadness, and surprise present the advantages of being meaningful to non-experts, many researchers use multi-dimensional models such as valence-arousal (Russell, 1980) or pleasure-arousal-dominance (Mehrabian, 1996) to classify emotions in a 2 or 3 dimensional space. Appraisal theories such as the Ortony, Clore & Collins (OCC) model (Ortony et al., 1990) or Ira Roseman's theory (Roseman, 1984), which explain emotion elicitation in terms of cognitive evaluations of significant events, are still rarely exploited in recognition and detection studies. As for the classification method, most works use approaches based on feature extraction and machine learning (e.g., Support Vector Machine) (Hovsepian et al., 2015), decision trees (Plarre et al., 2011), while solutions based on expert knowledge (e.g., rule-based) are more rare. Recently deep learning methods have been applied (e.g., convolutional deep belief networks, Ranganathan et al., 2016). The latter are, however, still limited by the capacity to collect a sufficient amount of data. EMSR methods might be user-dependent (or person-specific), built from the data of a specific user to detect his/her own emotions, or user-independent, built from the data of multiple users to detect emotions of any user. Regardless of the emotion theory and classification method used, one of the biggest challenges in EMSR consists in collecting and annotating data for both model creation and testing (Constantine and Hajj, 2012). In this paper, we address this challenge by providing a thorough discussion of existing methodologies for dataset creation as well as proposing evaluation criteria and tools to compare datasets and develop new ones. While most of EMSRs use similar methods to collect the affect related data, in this paper we focus on physiological signals.

So far, building physiological datasets for EMSR was usually performed in laboratory settings by purposely inducing emotions in subjects at specific time intervals. It allows experimenters to control the stimuli and reduce the number of contextual factors that may influence the subjects' reactions. To date, few studies have attempted to create real-life (not induced) emotions datasets, i.e., collections of affect-related data, outside of the lab, in reaction to everyday events. In the literature, the terms “in the wild” (Dhall et al., 2013), “in the fray” (Healey et al., 2010), and “in real-life” (Devillers et al., 2005) are used to describe such approach, in which the experimenters do not control the emotion elicitation process. In this methodology, the subjects can be, for example, monitored during their everyday activities over long time periods in order to collect their natural reactions. This kind of study can either be *ambulatory* (Healey et al., 2010) where people are able to move freely, or *static* where people experience real-life emotions but constrained to a specific location [e.g., a desk in a workplace (McDuff et al., 2012) or during an exam (Melillo et al., 2011)]. This similarity to real-life settings defines the ecological validity of a study (Ladouce et al., 2017).

There exist several potential applications of *the EMSR for “real-life applications”*, i.e., methods able to recognize emotions, moods or stress, in the wild (not induced, elicited by real-life events). Let us consider two examples of such an applications. The first example (Example 1) would be a smartphone mobile application designed to be a personal life coach. Such app would be able to detect the users' emotions helping them to become more aware of their own feelings, and to develop a more positive attitude toward life and healthy habits (Woodward et al., 2019). In this specific case, a EMSR module would be needed to recognize the users' real-life emotions, e.g., while they perform any activity, therefore, in an ambulatory setting. This EMSR model should be functional for any user desiring to acquire such a system.

The second example (Example 2) of such real-life application would be a system aiming at detecting real-life emotions for patients with Locked-in Syndrome (LIS). Such patients are unable to move any muscles beside the eyes and are unable to speak (Smith and Delargy, 2005). Consequently these patients would benefit from any system that allows them to communicate with the others, including the communication of emotions. For instance, the LIS patients were positive about the system enabling them to communicate explicitly emotions through a gaze-controlled system endowed of an avatar able to “display” the patients' emotions though facial expressions and voice. Such communication can be facilitated having an EMSR model working in-the-wild. In the case of building EMSR system for LIS patients, in theory there is no need to be concerned about ambulatory challenges. In practice, however, it might be difficult to find enough number of patients in this state to build a robust EMSR model, i.e., the model that works for every patient. Additionally, it might be unethical and difficult to involve such patients in long data collections and early testing stages of the model. In this case preliminary testing might be required with subjects without motor impairments, and, thus, probably performed in ambulatory settings. Only in the last step, such model would be adapted to the LIS patients.

In this paper, we take the perspective of the researcher or software developer who needs (1) to create a new dataset to be used for EMSR or (2) to build a EMSR model on top of existing dataset. We discuss the issues related to creation of “real-life application”—oriented datasets. We compare different data collection methods enumerating their advantages, challenges and limitations. In particular, we focus on physiological data collection outside of the laboratory as it represents a way to access people's emotional state without invading their privacy (e.g., using video, audio) and without being cumbersome (thanks to the size of the sensors). This paper presents a set of guidelines that may be used to build physiological datasets for EMSR. In order to facilitate the comparison and evaluation of such studies, we introduce a visual method to assess EMSR studies in terms of their ability to be used in real-life applications. This graphical method is used to visually compare the existing data collections. We then present an overview of the studies that take a step toward creation of EMSR based on physiological data collected outside of the laboratory.

While some reviews on emotion recognition from physiological signals exist (Jerritta et al., 2011) including systematic reviews (Kreibig, 2010; Wac and Tsiourti, 2014), the aim of this work is focused on showing the variety of methods and issues related to data collection in-the-wild. Therefore, we favored a broad-spectrum description of related works rather than a detailed list of stress-related datasets. Within this perspective, this paper can be of interest not only to experts in Affective Computing and Artificial Intelligence targeting development of new EMSR systems, but also researchers in Psychology. The comparative analysis of previous research related to data collection for emotion recognition in-the-wild is extremely important both for the design of future EMSR-based digital applications and for psychology research, as it highlights their strengths and weaknesses regarding their validity in real life situations. Furthermore, by analyzing previous studies from this point of view, the paper offers a sort of guideline for the design of novel experiments regarding emotions in the wild, which we believe can be valuable for researchers working on Emotion Science.

The main contributions of the paper are:

- While other recent surveys on EMSR make a census by considering expressive modality (e.g., El Ayadi et al., 2011; Kleinsmith and Bianchi-Berthouze, 2013), this work brings a new point of view to the field by focusing on methodologies for physiological data collection to build real-life EMSR applications in the wild.
- We propose a complete list of criteria as well as a novel graphical aid to compare and evaluate any existing and future affect-related datasets in terms of their applicability in real-life applications.

The paper is organized as follows: In section 2, we review existing data collection techniques by presenting emotion elicitation methods. In section 3, we present the in-the-wild methodology by discussing its advantages and disadvantages compared to traditional methods. In section 4, we present the categories and the visual aid chosen in this paper to assess the presented papers. Finally, in section 5, the existing studies on emotion, stress and mood recognition in the wild using physiological data alone or associated with other modalities are presented. Currently (1st July 2019) available commercial devices for ambulatory physiological data collecting are listed in the annex.

### 1.1. Related Works

Some works were published recently that survey emotion recognition from the physiological signals. Wac and Tsiourti (2014) provided systematic review of the wearable systems used to assess the affect in ambulatory conditions. The authors provide the deep analysis of the recently developed devices and propose also the list criteria for the device choice which include the study duration, number of participants, methods of ground truth assessments, whether the assessment can be performed online or offline, as well as compliance with technical standards.

Aiming at finding specific ANS responses for distinct emotional states (Kreibig, 2010) surveyed 134 empirical studies. In total 16 emotions (i.e., 6 basic emotions, 8 additional positive emotions, anxiety and suspense) were considered, and for each of them the authors sum up all observed ANS responses in different studies. Recently Shu et al. (2018) provided un updated survey of computational methods applied to physiological signals for emotion recognition In that work the authors listed different methods of emotion induction, capturing ANS signals, signal processing and feature extraction, fusion, and the large range of classification algorithms. Regarding surveys of existing datasets, Valstar (2019) reviewed recently existing multimodal databases, mainly focusing on audio-visual data.

Compared to above mentioned works, in this paper we focus more on the process of data collection as well the data annotation, and we try to provide a set of criteria to assess existing and future ones.

## 2. Common Emotion Elicitation Techniques Used for Data Collection

While the remainder of paper is focused on physiological signals, established techniques to elicit emotions are common for collecting other types of signals (Kory and D'Mello, 2015). We start this short survey by mentioning current techniques, in which there is no emotion elicitation protocol at all, as the participants do not actually feel any affective state but only pretend to react in an emotional way. These techniques often involve the participation of actors who act emotion expressions through facial expressions, body movements and speech (Wallbott and Scherer, 1986). Several researchers, however, claim that the spontaneous expressions of emotions are different from acted ones. For instance, Hoque et al. (2012) found significant differences in facial expressions of acted and induced emotions. Consequently, the EMSR models trained on acted data may not work properly in real-life applications. Using actors seems not to be viable for physiological signals collections as people may not be able to simulate their own physiological reactions. Actors may use some techniques such as the Stanislavski's method (Cole, 1995) to make their acting more natural. Other methods of self-induction of emotion have been used in scientific literature: e.g., in Vrana (1993) subjects are asked to apply the guided imagery method that consists in thinking about specific situations to elicit emotions. Retrospection is another commonly used techniques where participants are asked to narrate a story from their past when they experienced a given emotional state (e.g., Pasupathi, 2003).

Other studies on emotion, mood or stress try to induce more genuine reactions in their participants by using validated experimental protocols. These usually consist of exposing the subjects to some pre-defined and pre-validated stimuli for emotion induction. In such studies the experimenter has control over the environment such as the type, duration, order of the stimulus and the position of subject (e.g., whether he is sitting or standing). For instance, the widely used IAPS database (Lang et al., 2008) contains 956 images chosen to induce emotions and rated on valence and arousal by 100 participants. It was used in a great number of studies (Dikecligil and Mujica-Parodi, 2010; Fox et al., 2010; Schmidt et al., 2011; Walter et al., 2011). Other example—the Geneva affective picture database (GAPED) (Dan-Glauser and Scherer, 2011)—contains 730 pictures similarly rated. Also open-access annotated datasets of movie extracts exist. Some of them are explicitly created to induce specific emotional states in viewers, while others made for automatic analysis of affective content in a movie (by extracting some scene characteristics such as lightness, quantity of motion etc.). Independently of their primary aim, both types might be useful to evoke emotions in-the-lab conditions and collect the physiological data. The examples are the LIRIS-ACCEDE dataset (Baveye et al., 2015) containing continuous annotations of arousal and valence, and FilmStim (Schaefer et al., 2010) annotated with 6 discrete labels. Other more specific datesets include “the emotional film database for Asian culture” (Deng et al., 2017), which was validated through physiological measurements, or the E-MOVIE dataset (Maffei and Angrilli, 2019) composed of the movie extracts of fixed duration making them suitable for psycho-physiological research. Interestingly, the latter is annotated not only with emotion dimensions but also with discrete emotion labels.

Artwork is a greatly used tool for emotion induction. For instance, showing some extracts of well-known movies (such as The Pianists, Mr. Bean) was the method adopted by Soleymani and colleagues to create the MAHNOB-HCI dataset (Soleymani et al., 2011). Rooney et al. (2012) established that 3D movies have an enhanced capacity to create arousal and emotion when compared with traditional 2D movies. Within performing arts the physiological data of the performer can be also collected. For instance, Niewiadomski et al. (2017) and Lussu et al. (2019) explored whether the respiration signal captured by a standard microphone placed near to mouth can be used to classify the expressive movements. For this purpose, they collected the respiration data of dancers performing the sequences of expressive movements.

Music stimuli have also been used in a few studies (Kim and André, 2008; Konečni, 2008; Kreutz et al., 2008). They are often associated with other inputs such as light and storytelling (Kim et al., 2004). Similarly as for the visual stimuli, several audio based datasets have been created that can be used to generate affect-related physiological data collections. The examples include a dataset of 110 film music excerpts annotated with discrete labels, and arousal, valence dimensions (Eerola and Vuoskoski, 2011) used to induce emotions by Vuoskoski and Eerola (2011) and DEAM (Aljanaki et al., 2017). More recently it has been shown that also other artistic activities may convey emotional and physiological reactions in the wild e.g., watching the painting (Tschacher et al., 2012) and making the painting (Haiblum-Itskovitch et al., 2018). Thus, they can potentially be used for the data collection purposes.

Methods requiring active participation of subjects were also used, e.g., by using video games (Tognetti et al., 2010), virtual reality (Ververidis et al., 2008) and more recently immersive VR-games (Bassano et al., 2019). The latter uses a system composed of a VR-game and software platform to collect the player's physiological data. The data recordings are synchronized with the VR content presented to a player, so it is possible to trace which games events evoke specific physiological reactions. In particular, to evoke specific emotions, the VR—game was designed following the emotion elicitation process described by Roseman's appraisal theory (Roseman, 1984).

Annotating interfaces such as PAGAN (Melhart et al., 2019) or CARMA (Girard, 2014) (see section 3.3.2.2 for more details on annotation tools) have been developed to collect affect ratings by participants experiencing medias. However, researcher must be careful when using the movie or audio extracts as an emotion induction method, as it was found that perceived emotions by the observer (i.e., interpretation of the movie content, e.g., emotions of a movie character perceived by the observer) do not always agree with induced emotions (i.e., emotions felt by the observer)—both when using video (Muszynski et al., 2019) and audio (Kallinen and Ravaja, 2006).

Other less common emotion induction methods e.g., guided activation of specific facial muscles or postures (without being aware of corresponding affect) (Zajonc et al., 1989), can be found in the literature. These are based on facial feedback theories (Tomkins, 1962; Izard, 1977) according to which posing the facial expression, which corresponds to the specific emotion, may induce the corresponding emotional state in the person performing it. Researchers tried also to induce emotions by creating social scenarios in the lab simulating some realistic social interactions. For instance, Harmon-Jones and Sigelman (2001) asked the participants to write about an important subject to them, which was then negatively rated (regardless of the content) by a second participant. An aggressive comment and a low mark was expected to induce anger in the subjects. This type of study, especially the one focusing on negative emotions, usually requires that the participant is not aware of the experimental procedure. Niewiadomski et al. (2016) elicited expressions of amusement by having participants playing social games with their friends in the lab. Amodio et al. (2007) presents additional guidelines for building such scenarios such as a the elaboration of a credible cover story, a constant experimenter behavior and the conduct of post-experimental interviews.

Avatars, virtual agents and social robots have also been used to create highly controlled experimental social scenarios. The big advantage of using these technologies is that they can replace the human partner and be used to generate sequences of stimuli for multiple human participants in simulated social interaction scenarios. Meanwhile, the experimenter can maintain control over the stimuli generation (e.g., verbal and non-verbal behaviors of such the virtual agents/avatars/robots, timing, turn-taking etc.) and it the same procedure can be easily repeated it with a large number of participants. For instance, AlZoubi et al. (2012) used an avatar to induce boredom confusion and curiosity for expression detection. Turner-Cobb et al. (2019) measured the physiological reactions during a stressful task consisting of performing a mock interview in front of a robot audience. Shortcomings of this methodology should also be mentioned, including the fact that creation of realistic human-like non-verbal behaviors by artificial agents is still a significant challenge.

Other researchers have tried to collect spontaneous affective reactions while controlling the experimental environment by performing supervised real-life studies. These consist of putting the subjects into situations that usually bring about a strong emotional reactions e.g., sky-diving (Dikecligil and Mujica-Parodi, 2010) or driving in difficult conditions (Healey et al., 2005).

To introduce stress, additional techniques are available (Karthikeyan et al., 2011). The Stroop test from 1935 (Stroop, 1935)—presenting words representing a color written in a different color and asking to verbally state the written color—have been used in many studies (Pehlivanoğlu et al., 2005; Zhai and Barreto, 2006). Hassellund et al. (2010) used a cold stressor, which consists in immerging one's hand in cold water. Other popular stress induction stimuli include, for instance, performing mental arithmetic exercises (Ring et al., 2002), voluntary hyperventilation (De Santos Sierra et al., 2011), public speaking (Von Dawans et al., 2011), or computer games (Rani et al., 2002).

The previously presented techniques all have their own set of advantages and limitations. They will be further discussed in comparison with the in-the-wild methodology in next section.

## 3. The In-the-Wild Methodology

### 3.1. Why Are Datasets In-the-Wild Needed?

A large number of studies on automatic emotion recognition from physiological signals obtained good recognition rates (Jerritta et al., 2011) but very few of the proposed methods were then tested on data collected in the wild. Their applicability in real-life applications is therefore not confirmed.

Wilhelm and Grossman (2010) presented the risks of such approaches in terms of physiological signals, comparing laboratory induced stress and the ones occurring in ecological settings. They studied the case of physiological reaction to stress and compared laboratory induced stress to real-life ones such as watching a soccer game. They found the heart rate during the latter was elevated significantly compared to the former. Similarly, Xu et al. (2017) considered the validity of using in the lab collected data for ambulatory emotion detection. Their findings suggested that EDA, ECG, and EMG greatly differ between real-life and laboratory settings and that using such methodologies result in low recognition rates (17–45%). Thus, it is necessary to validate EMSR methods in the wild to be able to automatically recognize people's emotional states in real-life applications, such as the ones introduced in section 1. Additionally, even if emotion laboratory induction techniques use a highly controlled experimental procedure there is no certainty that the subjects will actually experience the desired emotion. Indeed, people can react differently to the same stimuli (Kret and De Gelder, 2012). For instance, one person might enjoy horror movies and find the experience entertaining, while someone else might find it scary and stressful. This might also be the reason why common passive methods of emotion induction, e.g., image datasets (see section 2) usually focus only on small subsets of “basic” emotions or arousal-valence dimensions. More subtle or complex emotions (e.g., guilt, pride) are probably more person-specific.

Furthermore, it is known that people's physiological signals adapt with age (Kostis et al., 1982) or fitness level (Melanson and Freedson, 2001). User-dependent EMSR systems may then need either to use adaptive models to include such changes or to allow the users to periodically re-train the model which may be difficult for models based on in-the-lab data (see section 3.2.3).

Using in-the-wild data for both the model building and testing phases brings additional advantages. Firstly, using in-the-wild data allows for iterative learning. By using data collected in the wild to build a model, it becomes possible to improve the models over time. This approach requires the use of in-the-wild data collection combined with self-reporting (see section 3.3.2.1).

Secondly, as mobiles phones and personal sensors become more and more popular, this data collection approach also allows the usage big data (Laurila et al., 2012) allowing the application of the latest techniques of data mining and deep learning. Indeed, model created from users self-report input and real-life emotions could allow for the collection of an extensive dataset. People are already reporting their emotion on mobile apps for the sole purpose of self-monitoring (e.g., “The Mood Meter”^2^, “Pixels—mental self awareness”^3^, “Mood diary”^4^). There is only a small step to associate such data labeling to physiological sensors using mobile applications. Preliminary work toward this aim was recently proposed in Larradet et al. (2019).

### 3.2. Advantages

In order to present the advantages of the in-the-wild methodology, we compare it with the previously presented techniques for data collection in the lab (see section 2).

#### 3.2.1. Ethical Issues

Inducing negative emotions such as anger or sadness can be problematic due to some ethical constraints. Usually only low intensity emotion induction methods such as IAPS images or movie clips (see section 2) are acceptable to Ethical Committees. The model would therefore not be able to learn from high intensity reactions as they would not be present in the collected dataset. On the other hand, real-life emotions collected using the in-the-wild methodology can be of any level of intensity and valence.

#### 3.2.2. Context

Although the creation of emotion elicitation procedures in the lab usually allows for a better control of the context (by minimizing unrelated factors that may influence the emotion elicitation process), several other factors may alter the affective reactions. For instance, some participants may already feel stressed or uncomfortable when participating in an experimental study in a laboratory (Britton et al., 1983). Emotions collected in the wild appear in a natural context without the presence of an experimenter to alter the subject's affects.

#### 3.2.3. Experimental Effort

Whether the data collection is performed in the lab or in the wild, an effort is necessary to build the dataset. In the laboratory, the experimenters need to prepare and validate the experimental protocol for emotion elicitation (e.g., trying interactive scenarios, preparing emotion induction games, finding appropriate images datasets, see more in section 2). In the wild, this effort is given to the subjects that need to report their emotions. In this case, no effort is required from the experimenter as the stressors/emotional situations are provided by life itself.

User-dependent EMSR models are often used in the case of physiological signals because of the important interpersonal differences in people's baselines and reactions to stimuli. Therefore, they tend to give better emotion classification results (Jerritta et al., 2011). However, for the reasons mentioned in section 3.1 an EMSR model may need to be updated after some time, and there is a need to reproduce the data collection and emotion elicitation process. For most emotion induction methods cited earlier (see section 2), it is difficult and probably ineffective to reproduce the method using the same set of stimuli. The previous knowledge of the stimuli may reduce or totally suppress the emotional reaction. Therefore a new set is then needed to repeat the emotion induction and data collection. It is, therefore, difficult to use an user-dependent induced emotions datasets to train EMSR models to be used over longer time periods, as it requires time consuming interventions (i.e., new stimuli preparation and new data collection) each time the EMSR model needs to be updated.

On the other hand, since user-dependent EMSR models built using the in the wild methodology only need self-reporting effort from the user and do not need any stimuli preparation. They can be updated, when the user requests it and agrees to self-report additional data. Consequently, this approach seems more suitable for real-life applications (e.g., the two examples mentioned in section 1).

### 3.3. Challenges and Limitations

#### 3.3.1. Absence of a Controlled Environment

In-lab data collection provides a controlled environment, that is similar for all the subjects. It allows for the comparison of many subjects' reactions to similar stimuli. Using a real-life dataset implies an unknown environment. The experimenter is unable to predict the emotional stimuli that will occur. Additionally, those stimuli will most likely be different for all subjects which makes inter-subject data comparison difficult. For instance, two subjects might experience happiness, but one due to an accepted scientific publication and the other because of a conversation with a friend. While both emotional reactions will be labeled as “happy,” they appeared in different contexts and are caused by different events. Because of this unpredictability and lack of a control over the data collection procedure, the experimenter is *a priori* unaware of the emotions felt by the subjects, and therefore this information needs to be determined a posteriori. Several ways of acquiring such information will be presented in the next section.

#### 3.3.2. Emotion Labeling

There are two main methods to acquire information about the data of affect-related events in an uncontrolled environment:

##### 3.3.2.1. Self-report

The most commonly used data labeling technique is controlled by the subjects themselves. In this method, participants are asked to report the time in which they felt an emotion, which emotion, and, eventually, some other parameters such as its intensity or context. This emotion self-labeling should be performed following specific emotion theory or framework. One can, for instance, report emotions using a set of discrete emotion labels (Zenonos et al., 2016), estimate valence and arousal (Carroll et al., 2013), or report significant potentially emotion-relevant events in terms of appraisals (Larradet et al., 2019). Each of these methods, brings challenges. For instance, it may be difficult for the subjects to estimate arousal as it is a concept that non-expert are usually unfamiliar with. Consequently their report might not be reliable. Indeed, Healey et al. (2010) found that subjects' valence and arousal reports did not correlate with their comments. They identified that subjects misunderstood the 2 dimensional map and interpreted the axis origin as 0 arousal instead of medium arousal. Techniques such as the SAM images (Bradley and Lang, 1994) are often used to make the self-reporting task more intuitive. Also, asking the subjects to self-report emotions by using labels such as “angry” or “sad” can also lead to problems. Indeed, Widen and Russell (2010) highlighted the need for a distinction between “descriptive definition” of emotion, as it is used in everyday life, and a “prescriptive definition”, as it is used by the scientific community. Similarly, the label understanding might differ within participants due to gender (Kret and De Gelder, 2012), or cultural differences (Mesquita et al., 1997). These differences might influence the quality of the dataset and, consequently, alter the capacity to automatically recognize emotions especially in the case of user-independent EMSR model. Larradet et al. (2019) addressed this problem and used an appraisal theory-based questionnaire to help the subjects provide precise information about the emotion elicitation events, without the need for them to pick a specific emotion label.

Oversight is another problem derived from subjects labeling their own data. One may not immediately report the felt emotion and then, simply forget to do it. Depending on the application, rating the emotion in terms of intensity might also be necessary. However, subjects might underrate their emotions for several reasons, e.g., they may not admit that they felt sad or scared. Additionally, emotion self-reports tend to be less valid when performed a long time after the experienced emotion (Mauss and Robinson, 2009).

Furthermore, user-given annotation of emotions beginning and end times might not be precise. Subjects will tend to give approximate times, making the exact data labeling more difficult. Instead of asking the subject to voluntarily report emotions when they feel them, some studies use technology-enhanced methods (e.g., smartphone apps, or sending emails) to prompt the user to report emotions at regular intervals. This method is often called Ecological Momentary Assessment (EMA) (Shiffman et al., 2008) or Fixed Time-Based strategies (Wac and Tsiourti, 2014). For instance, it is used in Plarre et al. (2011) to collect physiological signals and self-reports in a natural environment over a 2 day period. In this experiment, the phone app periodically prompted the user to complete self-report questionnaires on their stress levels and emotions being experienced.

It is not clear, however, what is the optimal frequency of such prompting. Plarre et al. (2011) mention that their app prompted the users 25 times per day on average, however, asking too often can easily become bothersome to the subjects and therefore affect the quality of the self-reporting. Asking too rarely would increase the chance that the subject will report lower intensity of the emotion (Mauss and Robinson, 2009), or forgot to report. Schmidt et al. (2018) suggest performing an EMA every 2 h or five times a day coupled with the possibility to manually report emotions. When prompting regularly the subjects to self-report their emotions over certain period of the time (e.g., every hour), the collected information about the timing (i.e., when emotion started and ended) of reported states might not be precise. Thus, this technique may be more appropriate to collect information about moods which often have a longer duration (Mauss and Robinson, 2009), rather than emotions that are usually short (Gray et al., 2001). Indeed, Robinson and Clore (2002) states that increasing the time between two consecutive prompts increases the chances to collect semantic (related to beliefs and generalizations about oneself) memory of emotions instead of episodic (related to a particular event) ones. Accessing events details of the day may improve the recall (Lang et al., 1980; Robinson and Clore, 2001). However, retrospective thinking about too many details may disproportionately bias the emotional report (Kahneman et al., 1999). Asking subjects details about their daily lives might not meet the ethical regulations as it provides an easy way to recognize the subject. Asking the subjects to mentally reproduce the event without providing any information to the experimenter about it, might be a solution (Clore et al., 2001).

##### 3.3.2.2. Expert labeling

This method involves having one or several experts examine the data and use their knowledge and expertise to annotate emotions. This can be achieved using either the same physiological signal(s) as those that will be used in the EMSR model (Yin et al., 2006) or using a different type of signal (e.g., facial expressions, body movements). For instance, Healey et al. (2005) conducted an experiment where both physiological signals and video data were recorded in the wild. The video was analyzed by experts to validate the data labels given by the subjects and physiological data was used later to create an emotion detection model. However, this method often requires multimodal synchronized recordings which can be difficult to gather in-the-wild. The modalities which are most often used by experts when performing the annotation, such as video or audio, are usually the most intrusive.

Additionally, if more than one expert performs the annotation, they may disagree on perceived emotions. Consequently several methods were proposed to compute the inter-rater agreement and inter-rater reliability such as Cohen's or Fleiss's Kappa. The other approach may consist of a combination of expert labeling and user post-experiment cross-validation (Yin et al., 2006). Independent of the issues related to the collection of the synchronized video or audio data for the purpose of the expert annotation and labeling, several tools were created to help experts to annotate such data offline. Recent examples include PAGAN (Melhart et al., 2019) or ANVIL (Kipp, 2012). The first is an online platform for crowd-sourcing affect annotations of videos, and it incorporates three different one-dimensional techniques to be used for continuous annotation of affect dimension (e.g., valence and arousal). The second tool allows the researcher to define even very complex multi-layer annotation schema, that may include emotion labels and dimensions, but also expressive behaviors (e.g., facial expressions or gestures).

#### 3.3.3. Context

The other issue linked with emotion labeling is the amount of information not given by the subjects. For instance, a study might focus on two emotions: happiness and anger, and therefore the researchers within the data collection protocol may ask the participants to report only the events related to these two particular emotion labels. However, the subjects might still experience a much larger range of emotions during the data collection. At the same time, they might also perform unrelated actions such as smoking or drinking which may not be in the scope of the data collection and therefore would not be reported by the participants. These other emotions or actions, which remain unreported, may eventually corrupt the quality of the data labeling as they have an impact on the studied signal. For example coffee intake can affect Heart Rate (Green and Suls, 1996), and although progress has recently been made to reduce the effect of physical activity on emotion classification from physiological signals (Heinisch et al., 2019), still it is recommended to report such activity in self-reporting. For this reason, Schmidt et al. (2018) recommend collecting in parallel information about the physical activities and the sleep quality of the subjects and to conduct data-driven screenings interviews with the participants to gather additional context information. In their survey, Wac and Tsiourti (2014) discussed several other contextual factors (in the original paper they are called “confounding factors”) that may influence the accuracy of the monitoring of the affective states. These include traits, which are constant or slowly evolving during the life, being at the same time important factors contributing to interpersonal differences, such as gender or diseases (e.g., allergies), as well as instantly changing ones, e.g., metabolic body activity related to eating, cognitive load, posture of the person. Some other contextual factors are related to the social context, e.g., being alone or in company and to the environment, such as temperature and humidity which may also influence the captured signals. In particular, social setting has a great impact on induced emotions as highlighted by Muszynski et al. (2018) who studied the synchronization of affective responses of people watching movies together.

Consequently there is a need for experimenters to request additional reports from participants about factors that are known to affect physiological signals such as alcohol, coffee or drug intake, physical activity etc. Unfortunately, this also greatly increases the complexity of the study and may affect the willingness of the participants to follow the protocol. Other solutions might need to be considered such as embedded cameras or microphones to collect the contextual data.

#### 3.3.4. Ambulatory Systems

When it comes to real-life dataset collection, there is a distinction to be made between ambulatory and static studies. Collecting of real-life emotion data often requires long-term studies during which people can freely change location. This means that ambulatory systems are needed to collect physiological signals while the person is moving. Some existing studies do focus on real-life emotions felt by the subject, but the data collection was confined to a specific physical space, e.g., to a desk space (Roseway et al., 2015). These types of studies will be referred to as “static studies” (as opposed to ambulatory ones previously mentioned).

In ambulatory studies, several issues need to be addressed. First of all, the devices recording the data must be both mobile and comfortable as they must allow the subjects to move freely for extended periods of time. This is the main reason why real-life data collections using HR or GSR signals are more common than using, e.g., EEG. There are a few devices available commercially (July 2019) for physiological signals-based ambulatory studies which are presented in the Supplementary Materials. Some researchers chose to develop their own devices (Wilhelm et al., 2005). While it is important to choose small sensors to ensure the wearability of the device, some sensors might be more affected by movement than others. For instance, to calculate HR, it is possible to use small PPG sensors, from which the BVP is read, the InterBeat Interval (IBI) calculated and the HR extracted. This technique is reliable but very sensitive to sensor movement (Pietilä et al., 2017). An alternative approach is to use an ECG. Chest ECG, while being much more invasive, provides more precise data which are less affected by movement (Ge et al., 2016). The choice between the two is therefore a compromise between wearability and accuracy. There are also techniques that can be used to improve the accuracy of the IBI calculated from PPG (Torres et al., 2016). The most common is the use of a 3D accelerometer to detect movement (Lee et al., 2010). Furthermore, HR is also greatly affected by physical activity (e.g., sports) and it is important to remove from the physiological data the periods of such activity. Once again accelerometer may help detecting such activities with some limitation. Novel technologies might allow the heart rate to be measured for emotion recognition without the need for any on-body device through the use of RF signals reflected off the participant's body (Zhao et al., 2016).

Neuroimaging systems (e.g., functional Magnetic Resonance Imaging—fMRI—and Positron emission tomography—PET) had demonstrated their potential value in investigating affective processes (e.g., Costafreda et al., 2008, for a meta-analysis on Amygdala activation during the processing of emotional stimuli). Salimpoor et al. (2013) used fMRI to observe how pleasure as aesthetic reward can arise from the interaction between mesolimbic reward circuitry (especially the Nucleus Accumbens) and cortical networks underlying the auditory analysis and assessment. Furthermore, it was shown that even emotion reappraisal activate various brain regions such as the fronto-parietal circuit including the parietal cortex, dorsolateral prefrontal cortex, supplemental motor area and the insula (Buhle et al., 2013). These findings are in line with fMRI data showing that both the perception (i.e., observing, listening) and expressions (i.e., motor actions, speech) conveying positive or negative mood and/or affect-related attitudes (e.g., gentle vs. rude) produce the activation of the insular cortex (Di Cesare et al., 2015, 2017).

However, even if neuroimaging can be effectively applied to the study of emotional processes (e.g., Sabatinelli et al., 2017), it can be difficult or even impossible, to adopt it in the study of affective phenomena in-the-wild, since it must be used in a laboratory or similar setting with high constraints in terms of subjects' mobility. However, for some studies e.g. paralyzed patients, this setup would of course form a common daily setting. To consider physiological measures with lower spatial resolution but higher temporal resolution, electroencephalography (EEG) and functional Near Infra-Red Spectroscopy (fNIRS) can also be adopted (Balconi et al., 2015) and these techniques do allow the person to walk and move in non-laboratory settings, wearing a portable sensorized headset. To ensure the ecological validity of the data collection in the wild such systems should not be visible to other interlocutors (e.g., they can be hidden under a cap). Exceptions can come from studies in which the everyday setting can be based on watching a television program or interacting with a computer, as in neuromarketing studies (Gkaintatzis et al., 2019) or neuroergonomics studies (Watson et al., 2019) especially in experimental paradigms of BCIs (Placidi et al., 2019).

Ambulatory studies tend to collect noisy raw data that must be processed before it can be used for emotion recognition. Several layers of processing might be required such as filtering (low-pass filters, smoothing filters and so on). The topic of post-processing is out of the scope of this paper but it is covered by previous survey papers (Jerritta et al., 2011).

#### 3.3.5. Long-Term Experiment

In an in-the-wild setting, it is unknown a priori how many times the subject will experience a certain emotion during the study or if he will experience it at all. However, some techniques exist to increase the likelihood of the emotion during the collection period. For instance, some subjects might know specific events in their future that are likely to trigger emotions (e.g., public presentation, important meeting, job interview). Studies involving multiple emotions might require subjects to experience a full range of emotions. This will however, greatly impact the length of the study. In that case, it is even more important to provide devices that are comfortable, so it would be acceptable for a subject to wear them over a long period of time. he possible length of the study. Indeed, the more comfortable the device, the more it would be acceptable for a subject to wear it over a long period of time.

#### 3.3.6. Lack of Datasets

Considering the great inter-person variability in physiological signals of emotions, it is important to work with data of a large number of subjects. For this reason, open access datasets are very valuable for EMSR research. Unfortunately, existing open access datasets (e.g., Dan-Glauser and Scherer, 2011; Koelstra et al., 2011; Abadi et al., 2015; Sharma et al., 2018; Markova et al., 2019) contain only the data of induced emotions.

## 4. The GARAFED Method

In this section, we propose a new assessment of the data collection methodologies based on their utility for building EMSR models for ambulatory real-life applications. Eight criteria were selected, each containing sub-categories. While other applications might have different needs and requirements (e.g., detecting stress during a written exam does not need an ambulatory setup), our assessment will be made considering exclusively any ambulatory real-life applications. In addition, even though other choices must be made when building EMSR models (such as emotion theory, see sections 2 and 3), they are not included in this assessment model. This is because such choices cannot be ranked from the most to the least suitable for real-life applications and they usually depend on the specific application.

For categories defined as intervals (e.g., between 3 and 7 days), the lower boundary (e.g., 3 days) is included in the category, and the higher number (e.g., 7 days) is not.

### 4.1. The GARAFED Categories

#### 4.1.1. Emotion Origin

As stated in section 2, there are many possible methods to collect the emotion data. The emotion may be induced by an experimenter in the lab, or, in real-life, can be caused by other agents, events or objects (Ortony et al., 1990). By collecting data in situations similar to a natural setting, one may expect to obtain datasets which are more appropriate for EMSR for real-life applications (see our discussion in section 3.1). Here, we propose to classify existing methods into five categories:

1. Simulation of the emotion (e.g., acting).
2. Induction of emotions in-lab (e.g., movies, IAPS images).
3. Induction of emotions through supervised real-life activities (e.g., car driving, skydiving).
4. Real-life emotions, static monitoring.
5. Real-life emotions, ambulatory monitoring.

#### 4.1.2. Invasiveness

The size and portability of the system used to collect data in the wild impacts how easy it is for the subjects to carry it for long periods and thus the possibility to conduct longer experiments. This invasiveness factor has been separated into four categories:

1. Non-portable: the system needs to be linked to a power supply and/or require the experimenter intervention, such as sampling of salivary cortisol level.
2. Portable and highly invasive: the system is heavy bulky or invasive. It may include sensors such as nasal respiration sensors. It is not possible to wear it for many hours a day without discomfort for the subject. (ex: Vu-ams, De Geus and Van Doornen, 1996).
3. Portable and slightly invasive: The system is light. It can be worn for several hours a day but it is noticeable and/or potentially uncomfortable for the subject after a certain time (e.g., Shimmer3 GSR+ Unit).
4. Portable and non-invasive: The system is light and does not have an impact on everyday activities, even if used over long periods. It is similar to a commonly worn object such as a watch, a belt etc.

#### 4.1.3. Privacy

The input data used to classify emotions can infringe the privacy of the subject. Indeed, data such as video, voice or activities in calendar app would give the experimenter access to personal data. They may also allow for the identification of the subjects. While infringing the privacy does not influence the quality of the EMSR model, the use of such data usually is restricted or ethically unacceptable.

While this review focuses on physiological data that are less intrusive for the privacy than the data collected from other modalities, we also consider multimodal approaches (see section 5.2), which may be more intrusive. In this review we classify papers using the two categories:

1. Intrusive data: personal data or data that allows for identification.
2. Non-intrusive data: non-personal and does not allow for identification.

#### 4.1.4. Number of Experimental Days

Collecting data over many days increases the probability of gathering data covering a variety of situations and contexts. This variability may improve the robustness of the model. Wac and Tsiourti (2014) emphasizes on the difficulty to collect physiological signals in the wild, and in particular, the choice of the study length, that should be a compromise between collecting representative samples and limiting the burden for the participant. We aggregated the number of days used for the dataset collection process for each paper present in this review proposing an EMSR model (see sections 5.1.1.1, 5.1.1.2, 5.2.1.1, 5.2.1.2). By extracting 4 quartiles on this data, we defined the following categories:

1. Less than 3 days.
2. Between 3 and 7 days.
3. Between 7 and 34 days.
4. 34 days or more.

For papers that give a range to the number of days in the experiment (e.g., 4–6 days), the maximum time was taken (e.g., 6 days).

#### 4.1.5. Number of Hours per Day

The number of hours for data collection per day also greatly impacts the value of the dataset, and indeed, physiological signals may also vary with the time of day (Gjoreski et al., 2017). Here again we used the studies presented in this review to extract the quartiles that define the following 4 categories:

1. Less than 4 h per day.
2. Between 4 and 8 h per day.
3. Between 8 and 16 h per day.
4. 16 h a day or more.

For papers providing only a time interval per day (e.g., 12–14 h per day) the maximum time was taken (e.g., 14 h).

#### 4.1.6. Number of Subjects

As previously stated, high inter-personal variability is often observed in physiological signals of emotions. In order to create and validate an user-independent EMSR, it is usually recommended that the data is collected from many subjects. As in the previous sections, we used the quartile method to define the following four categories:

1. Less than 6 subjects.
2. 6 to 12 subjects.
3. 12 to 24 subjects.
4. 24 subjects or more.

Quartiles were averaged to the largest round number.

These criteria represent a data collection paradigm that can be: (1) used to build, or (2) used in the selection of a dataset to build, an emotion recognition model that is usable in ambulatory real-life applications, e.g., Example 1 presented in section 1. Ideally, the data collection would be done using non-invasive and non-intrusive sensors. A study of this type should be done for an extensive time with a large number of subjects. It is worth to notice that some of criteria discussed in this section, were also postulated by other researchers (e.g., Wac and Tsiourti, 2014; see section 1.1).

### 4.2. The GARAFED Visual Aid

**Figure 1:**
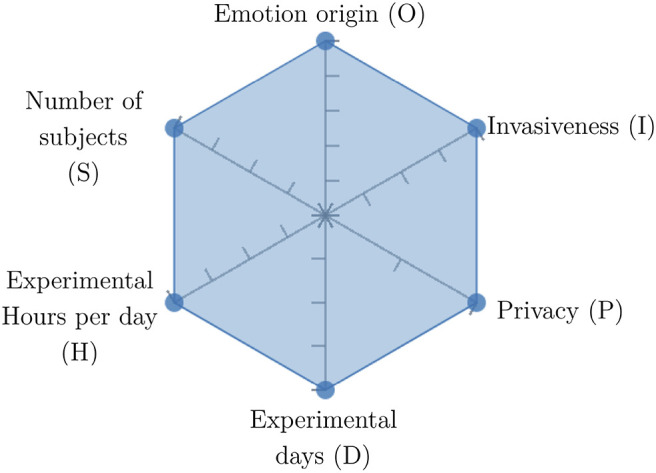
The GARAFED method.

In order to ease the assessment of existing and future works, we propose a open visual aid: the GARAFED (Graphical Assessment of Real-life Application-Focused Emotional Dataset) method (Figure 1). Inspired by the Adapted ECOVAL framework (Labonte-LeMoyne et al., 2018), it allows for the comparison of different datasets and evaluation of their utility when applied to real-life EMSR applications.

GARAFED is *open* visual aid tool, in the sense it can be extended (see our discussion in section 5.3) and possible extensions may include the inclusion of the contextual data e.g., the annotation of person-related or environment-related factor (Wac and Tsiourti, 2014, see section 3.3.3). However, we understand that most features like these factors cannot be expressed by a position along an ordinal scale. they can however be listed as a set of checkboxes to define the presence or the absence of a certain factor within each data collection plan.

GARAFED can be considered a tool for the affective systems designers who need reality-based datasets for generating their own computational models to recognize certain emotional states for their own applications (Aranha et al., 2019). These models are critically important to enable artificial systems with the capability to react appropriately to the affective changes in the human experience within a specific context where the model must be initially forged through an ecologically valid data collection approach.

Some exemplary applications that can exploit GARAFED-assisted data collection to generate reality-based models of emotion recognition are listed below:

- GARAFED can assist the definition of emotion origin to design the affective system underlying a virtual reality setups for stimulating emotional states to improve mental health, relationships, well-being, empathy (Schoeller et al., 2019);
- GARAFED can show the effectiveness of a certain amount of time (days and hours per day) to collect data for implementing a wearable solution assisting daily habits meditation and physiological self-regulation through biofeedback (Choi and Ishii, 2020) to treat also psychological issues like anxiety;
- GARAFED can help to select the appropriate number of subject for developing interactive systems (e.g., games, Larradet et al., 2017) designed to be controlled by people with rare conditions like Locked-In Syndrome (LIS, a condition where severe motor impairments spares sometime only the ocular muscles, as in the late stages of Amyotrophic Lateral Sclerosis, ALS) through a relax-based biofeedback that can be enriched by explicitly emotional features monitoring;
- GARAFED can be used to implement ethically adequate choices in data collection for investigating collective emotions (Skowron et al., 2017) in online social contexts without disclosing the affective state of each participant;
- GARAFED can integrate existing approaches to design training systems for professionals that must keep their mental focus during risky operations (as in the augmented reality neurotraining for surgeons in Barresi et al., 2015) in which emotional states can be critical but a low level of invasiveness of the physiological sensors must be maintained for avoiding any invasiveness in the simulative scenario.

## 5. Assessment of Existing Datasets

In this section, we study prior works that involved experimentation in real-life or supervised real-life environments. To build this corpus of studies we used combinations of the following keywords in Google scholar: “emotion,” “emotion recognition,” “emotion classification,” “emotion detection,” “valence,” “arousal,” “affect,” “in the wild,” “in the field,” “in the fray,” “in real life,” “ambulatory,” “physiological signals,” “biosignals,” “heart rate,” “HR,” “galvanic skin response,” “GSR,” “electrodermal activity,” “EDA,” “skin Conductance,” “SC,” “photoplethysmogram,” “PPG,” “blood volume pressure,” “BVP.”

Although the GARAFED may be applied to different types of input data, in this section we use it to assess papers focusing on physiological signals^5^. Here, we distinguish:

- Works using solely physiological signals (see section 5.1),
- Studies collecting physiological signals and additional inputs such as audio or video (see section 5.2).

In both cases, research papers will be separated into 3 categories:

- Studies which consists of collecting the data and proposing EMSR models in-the-wild.
- Empirical studies exploring physiological signals of emotions, mood or stress collected in real-life settings without proposing a detection or classification method.
- Studies which apply existing EMSR models or/and previous research results in specific real-life applications.

Only papers belonging to first category will be assessed using the GARAFED method as they provide the description of the data collection. The second category contains results that may be helpful for future model development. The third category, show EMSR real-life applications. Consequently the two last categories are a collection of relevant papers that might be useful to the reader interested in physiological data collection and EMSR.

Features extracted from the raw signals are also presented, as they are often used to improve EMSR machine learning algorithms. Common features of HR used for emotion recognition include for instance HRV, RMSSD, pNN50, or SDNN, and common features of EDA include for instance slope of the FDA. While additional signal processing such as Fourier Transforms or Wavelet transforms and feature reduction techniques such as Sequential Forward Selection (SFS) might be necessary to only select emotion related signals, they will not be presented in this review. More details on data processing, feature extraction and feature reduction can be found in Jerritta et al. (2011) and Shu et al. (2018).

A list of currently (July 2019) available devices to perform ambulatory studies is provided as a part of the Supplementary Materials.

### 5.1. Physiological Signals-Based Studies

#### 5.1.1. Datasets for In-the-Wild Detection and Classification

##### 5.1.1.1. Studies on stress

A few studies propose methods to estimate stress in real-life settings. Plarre et al. (2011), Hovsepian et al. (2015), and Gjoreski et al. (2016) trained a model with 21 participants in the laboratory and tested it in real-life settings with 17, 20, and 5 subjects, obtaining 71%, 72%, and 92% accuracy. Using a different approach, Dobbins et al. (2018), Muaremi et al. (2014) and Hernandez et al. (2011) used data from 6, 10, and 9 participants collected in-the-field to estimate stress obtaining 70%, 73%, and 78% accuracy respectively. Other researchers such as Healey et al. (2005) and Rigas et al. (2011) limited their works to supervised environments who aimed to detect stress in drivers obtaining respectively 97 and 82% accuracy. Similarly, Melillo et al. (2011) used a real evaluation from a university to collect data from 42 students estimating stress with an accuracy of 95%.

Lamichhane et al. (2016) monitored subjects for 5 days and addressed inter-individual differences using a Stress Response Factor in order to improve stress recognition models. Can et al. (2020) compared the machine learning models on laboratory data and on daily life data. When the models were trained the data in-the-lab, the accuracy of the system when tested in-the-wild improved significantly reaching 74% detection rate. Vila et al. (2019) estimated stress of travelers reaching an accuracy range from 92 to 100%.

Table 1 summarizes the datasets used in these studies and presents their respective GARAFED.

**Table 1:** Studies that collect the physiological data only and focus on stress.

**Authors**	**Signal**	**Emotion Labeling**	**Testing method**	**Affective states**	**User dependency**	**Accuracy**	**Approximate duration**	**Number of subjects**	**Extracted physiological features**	**Graphical representation**
Plarre et al. (2011)	ECG Resp Acc	Public speaking period	self-report Smartphone 25 EMA/day	Psychological stress	UID	Psychological stress: 90%	2 days 12-14h/day	Lab: 21	ECG: RRI, LF, MF, HF, LF/HF	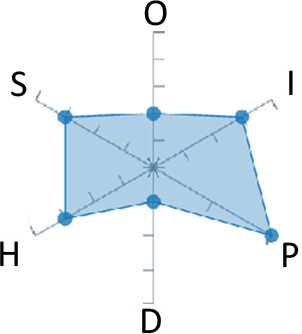
		Mental arithmetic								
		Cold pressor Self-report		Perceived stress		Perceived stress: lab :72% field: 71%		Field: 17	Respiration: ID, ED, RD, IE ratio, stretch, min Ve/min Vo, RSA	
Hovsepian et al. (2015)	ECG Resp Acc	Public speaking period	Self-report Smartphone 15 EMA/day	Stress	UID	lab:89% field: 72%	7 days 10–16h/day	Lab: 26 Field: 20	ECG: RRI, HRV, LF, MF, HF, LF/HF, HR	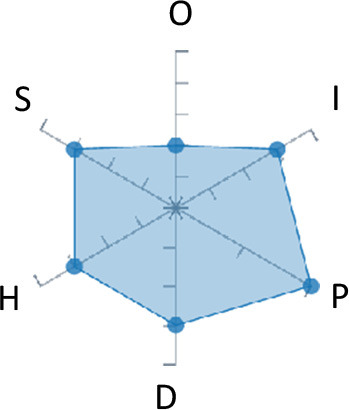
		Mental arithmetic Cold pressor Self-report							Respiration: ID, ED, RD, IE ratio, stretch, RSA	
Healey et al. (2005)	ECG EMG EDA Resp	Driving (rest, highway, city) Validated by: Self-report Score derived from video	Leave-one-out	Stress	UD	97.40%	1-7 days 2h/day	9	EKG: HR, RRI, HRV	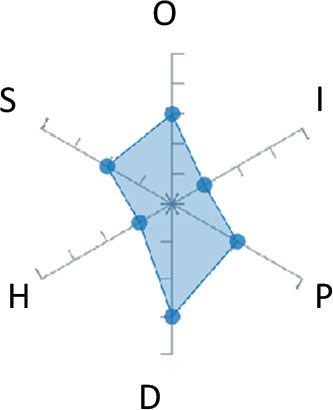
Rigas et al. (2011)	ECG EDA Resp	Driving Self-report voluntary oral	Leave-one-out	Stress	UID	82%	~40 days 50 min / day	13	ECG: RRV, HRV EDA: SCL, SCR, FAD, normalized measure of the differences Respiration: spectral entropy	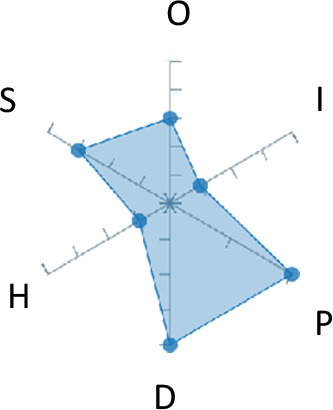
Dobbins et al. (2018)	PPG GSR	Self-report Smartphone 2/day	Leave-one-out	Stress	UID	70%	10 days Waking hours	6	-	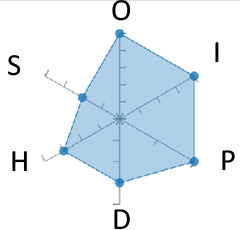
Gjoreski et al. (2016)	PPG GSR ST Acc	Mental arithmetic	Self-report Smartphone 4-6 EMA/day	Stress	UID	92%	55 days	Lab: 21 Field: 5	PPG: HR, HRV, RMSSD, SDNN, RRI, LF, HF, MF, 2 LF/HF, pNN20-50-70 GSR & HR & ST: slope, intercept of signal GSR: Peaks, significant peaks	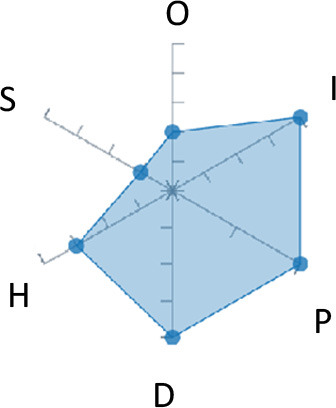
Muaremi et al. (2014)	ECG Resp ST GSR Acc	Self-report 1 / day	Leave-one-out	Stress	UID	73%	18 nights ~6h30/night	10	ECG: HRV, LF/HF, SD1/SD2 ST: Peaks	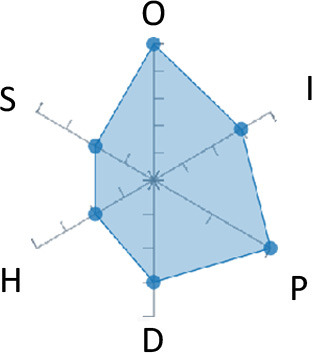
Hernandez et al. (2011)	EDA	Self-report 1 / call	Leave-one-out	Stress	Both	UD :78.03% UID: 73.4%	7 days work hours	9	-	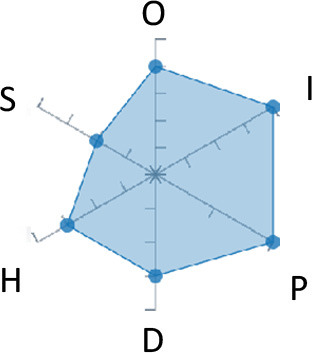
Melillo et al. (2011)	ECG	Stressor: University evaluation Control: After holidays	Leave-one-out	Stress	UID	95%	2 days 5m/day	42	ECG: HRV, RRI, SDNN, ApEn	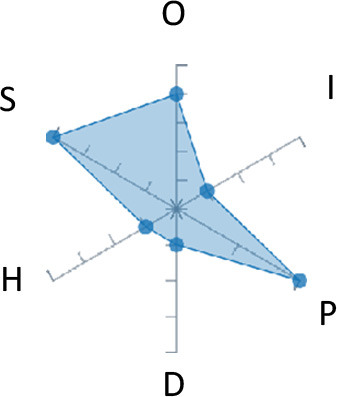
Lamichhane et al. (2016)	ECG 3D Acc Resp GSR	self-report Smartphone EMA every 30 min	Leave-one-out	Perceived Stress	UID	Average mean squared error by up to 32%	5 days work hours	10	ECG: RRI, HR, rmssd, LF, HR, LF/HF, pnn50, apen, sd1, sd2, sd1/sd2 GSR: scl, scp, scrr, scdiff2 Respiration: RR, cycles/m	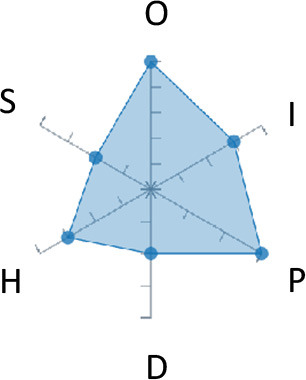
Can et al. (2020)	PPG GSR ST 3D Acc	Public speaking period	self-report Smartphone 1 EMA every 3h	stress	UID	73.8%	7 days 12h	14	EDA: Peaks Strong peaks PPG: HRV, RRI, SDNN, RMSSD, Pnn50, TINN, LF, HF, LF/HF, HRV triangular index	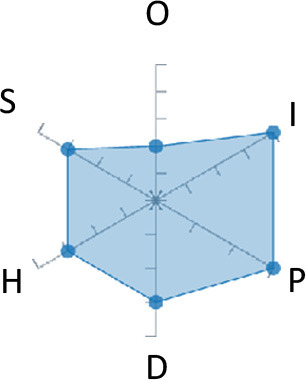
Vila et al. (2019)	PPG GSR ST 3D Acc	self-report	Leave-one-out	stress	UD	92.6% - 100%	3 days Waking hours	1	EDA: SCL, SCR Local minima PPG: IBI, RMSSSD, HR, LF, HF, LF/HF	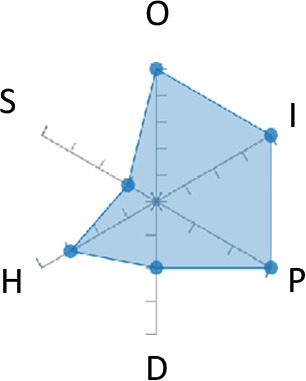

##### 5.1.1.2. Studies on emotions and moods

There are many fewer studies focusing on emotion or mood recognition methods tested in-the-wild. Carroll et al. (2013) studied emotional eating by detecting mood using a dimensional method. They reached 75% recognition for arousal and 72.62% for valence. Zenonos et al. (2016) focused on recognizing moods in work environments. They proposed a model that had an accuracy of 70%. Finally, Healey et al. (2010) studied emotion recognition in the wild with 19 participants and achieved an accuracy of 85% for arousal and 70% for valence. Schmidt et al. (2019) highlights the difficulties of in-the-wild emotion recognition in his field including 11 healthy subjects for 16 days. Their new methodology including multi-task CNN succeeded to reach a F1 score 1.8% higher compared to classical methods, however still relatively low (45%). Table 2 presents those studies as well as their GARAFED representation.

**Table 2:** Studies that collect the physiological data only and focus on emotions and moods.

**Authors**	**Signal**	**Emotion labeling**	**Testing method**	**Affective states**	**User dependency**	**Accuracy**	**Approximate duration**	**Number of subjects**	**Extracted physiological features**	**Graphical representation**
Healey et al. (2010)	GSR ECG Acc	Self-report Voluntary Smartphone 2D map	Self-report Smartphone	Valence-Arousal	UID	85% Arousal 70% Valence	5 days 8+ h/day	19	ECG: HR, HRV, RMSSD GSR; slope, kurtosis, Peak frequency, rise/falls times	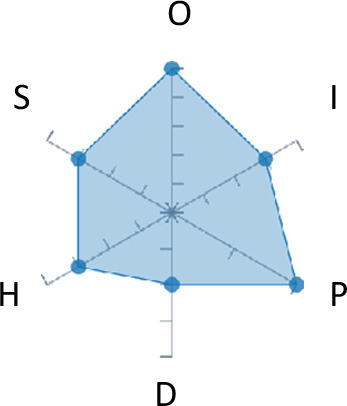
Carroll et al. (2013)	Resp EKG EDA Acc	Self-report Smartphone 1 EMA/h 2D map	Leave-one-out	Valence-Arousal	UID	Arousal : 75% Valence : 72.62%	4 days 4-6 h/day	4	EKG: HR	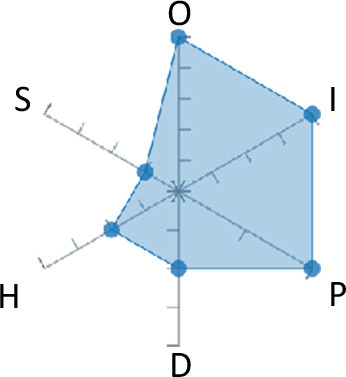
Zenonos et al. (2016)	ECG PPG ST Acc	Self-report Smartphone 1 EMA/2h Emotions	Leave-one-out	Excited Happy Calm Tired Bored Sad Depressed Angry	Both	Average : UD: 70% UID : 62%	5 days 8h/day	4	EKG: IBI, SDNN, RMSSD, pNN50, HRVi, TINN, PWTT, PSD, LF, HF, LF/HF	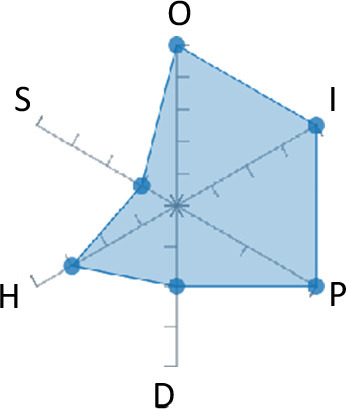
Schmidt et al. (2019)	ECG PPG ST Acc	Self-report Smartphone 1 EMA/2h & Voluntary	Leave-one-out	Valence & arousal State-Trait Anxiety Inventory Stress	Both	UID F1scores: 31% - 47%	16 days 15h/day	11	PPG: HR, HRV, EDA: Peaks	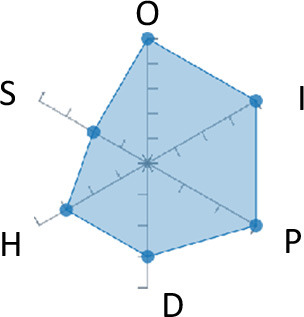

#### 5.1.2. Empirical Studies in Real-Life Environment

##### 5.1.2.1. Studies on stress

Most studies on stress in the wild are preliminary. They report findings and observations of physiological reactions to natural stressors without proposing a detection model. The disparities in stress experiences in the lab compared to in the wild are assessed by Dikecligil and Mujica-Parodi (2010). They compared HRV obtained from 33 subjects during 2 short term laboratory experiments (using IAPS images), a long-term hospitalized monitoring study (24h) and a supervised real-life study (180 min including a first-time tandem skydive). They found strongly predictive correlations between laboratory results and supervised real-life study. Similar supervised real-life studies were conducted, notably by Fenz and Epstein (1967), that monitored HR and respiration in 10 novice and 10 experienced parachutists during a jump. They found a sharp rise in physiological activity in novice jumpers and an inverted V-shaped curve in experienced ones. Wilhelm and Roth (1998) similarly studied HR and respiration during a plane trip with flight phobics which pointed additional HR as a reflection of participants anxiety. Kusserow et al. (2012b) monitored people in the wild as well as a musician, an Olympic ski jumper, and a public speaker. They found correlations between HR and stress. Baek et al. (2009) evaluated stress in driving using a custom car equipped with sensors (ECG, GSR, Resp). In this supervised real-life study, temperature, noise, time of day (night vs. daytime) and simultaneous arithmetic calculations separated were altered to create stressful environments. They found meaningful changes in physiological signals in simulated stressful environment. Different physiological reactions in participants were obtained for the same stressor. This highlights individual differences in reaction to emotional triggers.

Ambulatory in-the-wild studies were also conducted. Verkuil et al. (2016) proposed an in-the-lab calibration using rest, standing, cycling and stair climbing to improve the capabilities of categorizing metabolic and non-metabolic HRV reductions in-the-wild (24 h) using ECG and 3D accelerometer. HRV was found associated with negative affect and worrying. Johnston and Anastasiades (1990) studied the relation between HR and stress, arousal and time pressure in real life over a period of 24 h with 32 subjects. No significant relations were found between the HR and the emotional state in most participants. A significant relationship was obtained only in a small subset of subjects who were found to be more anxious, angry and with higher systolic blood pressure.

##### 5.1.2.2. Studies on emotions and moods

Studies on mood and emotions are less common than those focusing on stress. Myrtek and Brügner (1996) studied ECG associated with an accelometer to compare the data of laboratory induced emotional events to real life experiences. The self-reports of 500 participants during a 23 h ambulatory study were used and highlighted disparities between emotional arousal in-the-wild compared to results obtained in laboratory. Kusserow et al. (2012a) proposed an improvement to the additional heart rate method to determine arousal by improving the physical activity detection. They used this technique to assess arousal during daily activities such as taking public transport or office work. Picard and Rosalind (2000) proposed innovative ways to gather physiological signals for ambulatory emotion recognition, notably EDA sensors in earrings, shoes and glasses. Schmidt et al. (2018) collected 1081 EMAs from 10 subjects over 148 days.

#### 5.1.3. Real-Life Applications

##### 5.1.3.1. Studies on stress

While no gold standard in terms of stress detection in-the-wild exists, some studies have used the previously presented findings to assess stress levels for further purposes. For instance, Massot et al. (2011) used physiological signals to evaluate the stressful part of a walking path for blind test subjects, while Al-Fudail and Mellar (2008) used GSR to evaluate teachers' stress levels when using technological tools in the classroom. Similarly, Wettstein et al. (2020) studies teachers' stress using cortisol levels, HR, and HRV highlighting significant differences between free days and working days.

Myrtek et al. (1999) studied 29 blue and 57 white collar workers to determine stress and strain at work using HR. Several indices were used to define each type of strain: HR for total strain, physical activity for physical strain, and HRV for mental strain. Later, Myrtek et al. (2005) took the same approach to evaluate stress and strain in female students. They found that there are two type of persons “cool” (no emotion perception) and “emotional” (high emotion perception). Kimhy et al. (2009) evaluated the relation between stress and arousal for 20 patients with psychosis using both EMAs and the Life Shirt (Grossman, 2004) during 36 h ambulatory studies. Zhang et al. (2012), designed a mobile application that estimates stress using HRV and prompted the user to relax using breathing exercises. Rahman et al. (2014) studied stress in illicit drug users, daily smokers and drinkers. They used the previously mentioned model of Plarre et al. (2011) to assess stress and found after the first week a significant learning effect from the subjects in how to provide valuable data. Karlsson et al. (2011) studied the reaction of ambulance professionals to alarms. They showed that all subjects experienced increased heart rate when there was an alarm regardless of their experience, education, and gender, which implies the physical arousal is detected by the heart rate.

##### 5.1.3.2. Studies on emotions and moods

Existing models were often applied in real-life applications and experiments. For instance, Kim and Fesenmaier (2015) used EDA to estimate 2 travelers' emotions during a 4 days trip. Their mean EDA level seemed to correlate with their experience of each activity. Roseway et al. (2015) used EDA to determine arousal and HRV to determine valence in 10 participants during a 10 day study. Arousal was displayed using a color-changing emotional crystal to help mood-awareness during work in the workplace. The device seemed to improve stress control abilities in the subjects. Similarly, Snyder et al. (2015) used the color of a desk lamp to reflect subjects internal state estimated from EDA.

### 5.2. Multimodal Approaches

Collecting additional signals (e.g., audio) in addition to physiological signals might ease the recognition of emotions, moods and stress. In this section, we will present studies using a multimodal approach (physiological signals included).

#### 5.2.1. In-the-Wild Detection and Classification Studies

##### 5.2.1.1. Studies on stress

A few studies have used physiological signals combined with additional inputs to study stress. For instance, Muaremi et al. (2013) used smartphone information such as phone calls and calendar associated with heart rate to detect stress. They achieved a 61% accuracy. Rigas et al. (2011) associated driving event information with physiological signals to detect drivers' stress levels and obtained an accuracy of 96%. The summary of these studies and the assessment of their datasets may be found in Table 3.

**Table 3:** Studies that collect the multimodal data and focus on stress.

**Authors**	**Data**	**Emotion Labeling**	**Testing method**	**Affective states**	**User dependency**	**Accuracy**	**Approximate duration**	**Number of subjects**	**Extracted physiological features**	**Graphical representation**
Muaremi et al. (2013)	Microphone Acc GPS Phone calls Address book Calendar Battery ECG	Self-report Smartphone Audio 4 EMA /day	Leave-one-out	Stress	UD	61%	4 months 12-14h/day	35	ECG: RR, SDNN, RMSSD, pNN50, HRVi, TINN, ApEn, SD1, SD2, SD1/SD2, LF, HF, LF/HF	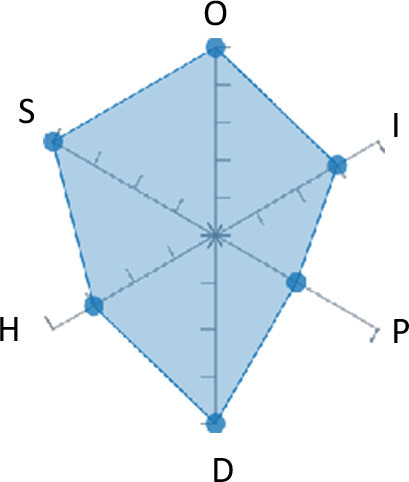
Rigas et al. (2011)	ECG EDA Resp Driving event	Driving Self-report voluntary Oral	Leave-one-out	Stress	UID	96%	~ 40 days 50 min/day	13	ECG: RRV, HRV, RRI EDA: SCL, SCR, FAD Respiration Spectrum energy Spectrum entropy	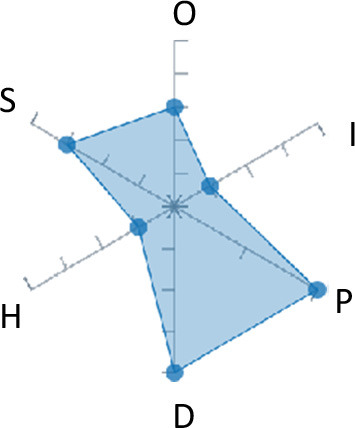
Gjoreski et al. (2017)	PPG EDA ST Acc Activity Hour of the day Type of day	Self-report Smartphone 4–6 EMA & Voluntary	Leave-one-out	Stress	UID	Recall :70% Precision : 95%	55 days	5	PPG: BV, HR, RMSSD, slope, power spectrum, LF,MF, HF, LF/HF, RMSSD, pNN20-50-70	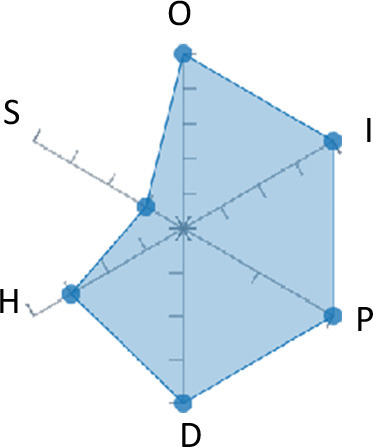

##### 5.2.1.2. Studies on emotions and moods

Moods and emotions have also been studied using multimodal inputs. Kanjo et al. (2018) associated noise environment, ambient light levels and air pressure to physiological signals to predict emotions with a 86% accuracy. Exler et al. (2016) used smartphone extracted data, such as calls and calendar associated with HR, to evaluate valence with a 91% accuracy. McDuff et al. (2012) limited their study to a work desk, adding devices such as cameras and position sensors. Their valence, arousal and engagement recognition model reached an overall accuracy of 68%. Those studies are presented in Table 4 alongside with their GARAFED assessment.

**Table 4:** Studies that collect the multimodal data and focus on emotions and moods.

**Authors**	**Signal**	**Emotion Labeling**	**Testing method**	**Affective states**	**User dependency**	**Accuracy**	**Approximate duration**	**Number of subjects**	**Extracted physiological features**	**Graphical representation**
Kanjo et al. (2018)	PPG EDA ST 3D Acc Air pressure Light GPS Noise	Self-report Smartphone SAM Constant evaluation	Leave-one-out	Valence	UID	86%	45 min	40	PPG: HR, HRV, Rmssd, PNN30-50, SDNN, HRV triangular index, spectral power, LF, HF, LF/HF	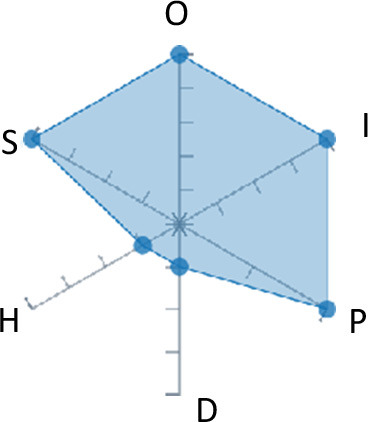
Exler et al. (2016)	Location Current app Microphone Messages Calls Light Connectivity Calendar Activity ECG	Self-report Smartphone Emotions % 1 EMA / h & 1 / specific event & Voluntary	Leave-one-out	Valence	UD	Avg: 68% Max: 91%	4 weeks Walking times	6	ECG: HRV, Hf, Lf, Lf/Hf, Pnn50, Rmssd, SD1, SD2, SD1/SD2, SDNN, SDSD	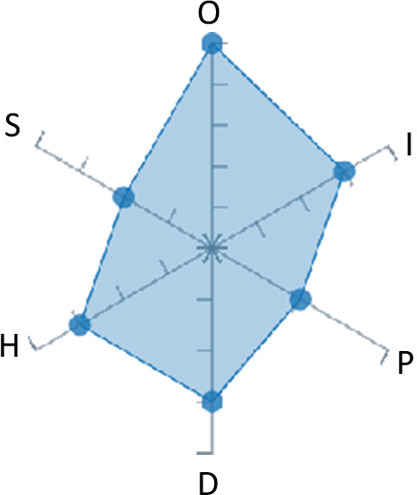
McDuff et al. (2012)	Video Posture Microphone EDA 3D Acc GPS File activity Calendar	Self-report 2D map EMA	Leave-one-out	Arousal Valence Engagement	UID	68%	2 days 10h /d	5	EDA: slope, different between first value and max, position of max, difference between value and min, position of min, zero crossings, peaks	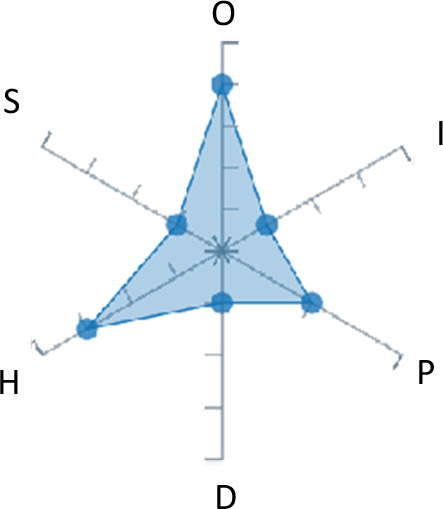

#### 5.2.2. Empirical Studies in Real-Life Environment

Pärkkä et al. (2008) studied the relationship between physiological signals, behavioral variables, exterior variables such as temperature, room illumination and self-reports of moods, and stress for 3 months with 17 subjects. Sarker et al. (2016) analyzed the GPS, activity and physiological data of 38 subjects during a 4 weeks experiment. They focused on the predictability of stress events duration and likelihood of stress events at different times of the day. They proposed a way of predicting the likelihood of a momentary stress episode esculating to become significant. Adams et al. (2014) collected EDA, microphone input and stress self-reports of 7 participants for 10 days. They found a correlation between audio profiles, EDA and self-reports of stress. Kocielnik et al. (2013) used GSR to evaluate arousal during a workday. The system created a 5-level arousal map (very high arousal to very low arousal) associated with calendar activities. 91% of the users found the generated arousal map a good reflection of their feelings.

### 5.3. Limitations

Whilst this method provides an intuitive tool for easy comparison of existing affect-related data collections in-the-wild, which can be understood even by non-experts, it does have some limitations. First, in this paper we have stressed that the context of the data collection is important, especially in the case of physiological data (see section 3.3.3). However, the GARAFED method does not introduce criteria to measure contextual information. Trying to add a metric to assess context would make the visual aid much more complex, as we are not aware of any method that could allow encoding the variety of contexts in a form of one additional dimension. The simple distinction between the “known-context” vs. “unknown-context” might not be enough. Future work in this topic should focus on appropriate classification of the variety of contexts under which the data collection is performed. The other factor that might influence the dataset assessment is the source of ground-truth (i.e., mainly through self-reporting methods). However it cannot be expressed by a position along an ordinal scale as there is no consensus which self-reporting method is the most appropriate one.

Another limitation of the GARAFED method regards the definition of the categories for criteria 4-6, which are based on the data provided from currently available datasets. If long-term physiological data collection becomes more popular, the proposed ranges might need to be updated.

## 6. Discussion

Accurate emotion recognition in-the-wild has a great potential to support affective science research and to develop applications designed for the general public. Whether it is applied to robotics (with robotics understanding of human emotions), to healthcare technologies (for monitoring the patients' affective state), to domotics (for adapting the home setting to the individual mood), emotion recognition has been a goal of the scientific community for decades. However, research has mainly been limited to laboratories and needs to be broadened to the wild to truly achieve meaningful progress. In this review we presented the main differences between classification and detection of emotions according to data collection in-the-wild and in the laboratory. We highlighted the main decisions to be taken, according to the goal of the desired study, their advantages, challenges and limitations, and we proposed a visual method—GARAFED—to categorize studies based on those main choices. Studies, past or future, using physiological signals or other types of input for emotion, stress or mood recognition may be assessed using this method. We presented the reason why there is a real need for research to be done in emotions recognition in-the-wild and showed that, while there has been some research in this area, there are still very few papers focus on this matter today. The quantified-self trend associated with the smaller and more portable sensor technology does, however, now make it easier for researcher to follow this path.

## Author Contributions

FL wrote the initial draft of the manuscript and defined GARAFED. RN contributed mainly to sections 1.1 and 2. GB contributed to the texts on ecological validity and neuroscientific methods, to the GARAFED representation of time and section 4.2. FL, RN, GB, DC, and LM contributed to manuscript writing, revision, read and approved the submitted version.

## Conflict of Interest

The authors declare that the research was conducted in the absence of any commercial or financial relationships that could be construed as a potential conflict of interest.

## Footnotes

^1^All abbreviations are explained in the Supplementary Materials.

^2^https://moodmeterapp.com

^3^https://play.google.com/store/apps/details?id=ar.teovogel.yip&hl=en_US

^4^https://play.google.com/store/apps/details?id=info.bdslab.android.moodyapp

^5^Accelerometers are widely used in combination with physiological signals, e.g., as an indicator of excessive movement and for filtering purposes. We therefore considered a study using physiological and accelerometer data as a physiological signals-only.
