# Supplementary material for: Toward Emotion Recognition From Physiological Signals in the Wild: Approaching the Methodological Issues in Real-Life Data Collection
Source: Front Psychol. 2020 Jul 15;11:1111. doi: 10.3389/fpsyg.2020.01111 (PMC7374761; doi:10.3389/fpsyg.2020.01111)
Supplement: Supplementary file 1 [file Data_Sheet_1.pdf]

frontiers

# *Supplementary Material*

## 1 LIST OF ABBREVIATIONS

| | |
|---|---|
| $Acc$ | Accelerometer |
| $AP$ | Airway pressure |
| $ApEn$ | Approximate entropy |
| $BP$ | Blood pressure |
| $BPC$ | Blood pressure cuff |
| $BVP$ | Blood volume pressure |
| $ECG$ | Electrocardiogram |
| $ED$ | Exhalation duration |
| $EDA$ | Electrodermal activity |
| $EEG$ | Electroencephalogram |
| $EMA$ | Ecological momentary assessment |
| $EMG$ | Electromyogram |
| $EMSR$ | Emotion mood and stress recognition |
| $EOG$ | Electrooculogram |
| $F$ | Force |
| $FAD$ | First absolute difference |
| $GP$ | General public |
| $GSR$ | Galvanic skin response |
| $GY$ | Gyroscope |
| $HF$ | High frequency |
| $HR$ | Heart rate |
| $HRV$ | Heart rate variability |
| $HRVi$ | heart rate variability triangular index |
| $IBI$ | Interbeat interval |
| $IBI$ | Interbeat interval |
| $ID$ | Inhalation duration |
| $L$ | Load |
| $LF$ | Low frequency |
| $MA$ | Magnetometer |
| $MF$ | Medium frequency |
| $PPG$ | Photoplethysmogram |
| $PSD$ | Power spectral density |
| $PWTT$ | Pulse wave transit time |
| $R$ | Research |
| $Resp$ | Respiration |
| $RMSSD$ | Root mean square of the successive diff. |
| $RRI$ | RR interval |
| $RSA$ | Respiratory sinus arrythmia |
| $RSA$ | R-R interval variability |
| $SC$ | Skin conductance |
| $SCL$ | Skin conductance level |
| $SCR$ | Skin conductance response |
| $SDNN$ | Standard deviation of NN intervals |
| $SP$ | Several products |
| $SpO2$ | Peripheral oxygen saturation |
| $ST$ | Skin temperature |
| $T$ | Torque |
| $TEMP$ | Temperature |
| $TINN$ | Triangular interpolation of RR intervals |
| $UD$ | User dependent |
| $UID$ | User independent |
| $Ve$ | Ventilation |
| $Vo$ | Volume |
| $W$ | Weight |

## 2 LIST OF DEVICES

Several devices are available for collecting physiological signals in the wild. Decision must be done regarding comfort, invasiveness and data accuracy. A list devices available for ambulatory data collection is given below with indications of their characteristics.

Companies with several products are marked with (SP). Invasiveness is marked according to categories defined in Section 4.1. It is specified for each device, if it was made for research (R) or for the general public (GP).

| Product (company) | Physiological signal | Other | Inva-siveness | Research / Gen. Public |
|---|---|---|---|---|
| E4 (Empatica) – SP | EDA, PPG, ST | Acc, GY | 4 | R and GP |
| Shimmer (shimmersensing) – SP | ECG,EMG, PPG, RESP, GSR | 3D Acc, 3D GY, 3D MA, L, W, F, T, P | 2-3 | R |
| Lifeshirt (VivoMetrics) | ECG, RESP, GSR, ST, PPG, EEG, EMG | Acc, TEMP | 2 | R |
| Biofeedback 2000x-Pert (Schuhfried) | EDA, PPG, ST, RESP | Acc | 2 | R |
| (movisens) - SP | ECG, EDA | Acc, Barometric AP, light, TEMP | 3 | R |
| (BITalino) - SP | Resp, PPG, ST, Glucose, BPC, SpO2, EMG, EEG, ECG, EDA | F, TEMP, Acc, Light | 1-3 | R |
| (somnomedics) - SP | RESP, EEG, ECG, EOG, EMG, ST, BP, SpO2 | TEMP, Light, Body position, Snoring | 1-3 | R |
| NeXus-4 (Mindmedia) | EEG, EMG, EDA, EOG, ECG, ST, RESP, BVP | | 2-3 | R |
| vu-AMS | ECG, RESP, EDA | Acc | 2 | R |
| (Vitamove) - SP | ECG, EMG, EEG, EOG , Resp | Acc, light | 2-3 | R |
| EQ02 LifeMonitor (Equivital) | ST, SpO2, GSR, BP cuff, PPG, ECG, RESP, | Acc, GPS, Body position | 2-3 | R |
| G. MOBIlab (Gtec) | EEG,ECG, EOG, EMG, Resp, GSR, ST, BP, SpO2 | Limb movements, Snoring | 2-3 | R |
| Mobile Impedance Cardiograph (Mindware Technologies) | EMG, RESP, ECG, GSR | Acc | 2-3 | R |
| Zephyr – SP | ECG, Resp, ST | Acc, time, location | 3 | R and GP |
| Feel | BVP, EDA, ST | Acc | 4 | P |
| Jawbone – SP | BVP, EDA, ST | Acc | 4 | P |
| eMoodmetrics | EDA | | 4 | P |
| Spire health | Resp | | 4 | GP and R |

**Table S1.** Available devices for ambulatory physiological signal collection
